# Breast cancer and social environment: getting by with a little help from our friends

**DOI:** 10.1186/s13058-016-0700-x

**Published:** 2016-05-26

**Authors:** Adam Hinzey, Monica M. Gaudier-Diaz, Maryam B. Lustberg, A. Courtney DeVries

**Affiliations:** Department of Neuroscience, The Ohio State University Wexner Medical Center, Columbus, OH 43210 USA; Department of Internal Medicine, The Ohio State University Wexner Medical Center, Columbus, OH 43210 USA; The Stephanie Spielman Breast Cancer Center, The Ohio State University Wexner Medical Center, Columbus, OH 43210 USA; 614 Biomedical Research Tower, 460 West 12th Avenue, Columbus, OH 43210 USA

**Keywords:** Social isolation, Breast cancer, Oxytocin, Catecholamines, Glucocorticoids

## Abstract

Social environment is a well-recognized determinant in health and wellbeing. Among breast cancer patients, inadequate social support is associated with a substantial increase in cancer-related mortality. A common explanation is that socially isolated individuals fare worse due to reduced instrumental support (i.e., assistance meeting the demands of treatment). However, the ability to replicate the detrimental effects of social isolation on mammary tumor growth in rodents strongly suggests an alternative explanation; i.e., socially isolated individuals have a physiological milieu that promotes tumor growth. This review summarizes the clinical and basic science literature supporting social influences on breast cancer, and provides a conceptual physiological framework for these effects. We propose that social environment contributes to the vast individual differences in prognosis among breast cancer survivors because social environment is capable of altering basic physiological processes, which in turn can modulate tumor growth. Appreciation of the role of social environment in breast cancer progression could promote the identification of patients at increased risk for poor outcomes. In addition, characterization of the underlying physiological mechanisms could lead to targeted disruption of detrimental pathways that promote tumor progression in socially isolated individuals, or exploitation of protective pathways activated through social engagement as novel therapeutic complements to contemporary treatments.

## Background: social influences on health: a focus on breast cancer

Social milieu dramatically affects the pathophysiology of a wide range of diseases. Nearly 25 years ago social isolation was identified as one of the most important risk factors for all-cause mortality [1], and these findings have been reaffirmed by multiple studies (reviewed in [2]). In addition, social isolation is specifically associated with decreased long-term survival following a diagnosis of various types of cancer, including breast cancer (BC) [3, 4]. In contrast, social integration is associated with lower overall disease-related mortality rates [5, 6]. There is encouraging concordance among clinical and basic science studies of social isolation on health outcomes, and several biological correlates have been identified. However, few of these studies have met the criteria for establishing a causal mechanism. Likewise, exploration of the physiological mechanisms through which social environment influences the trajectories of patients with cancer remains in its infancy. This review will provide a synthesis of the clinical and basic science studies demonstrating the effects of social environment on BC progression, and will offer a conceptual physiological framework to guide future studies aimed at exposing the biological underpinnings of this well-conserved phenomenon. The establishment of causal mechanisms linking social environment to tumor progression offers a unique opportunity to manipulate socially mediated biological pathways to enhance contemporary cancer treatments.

## Social constructs

**Fig. 1:**
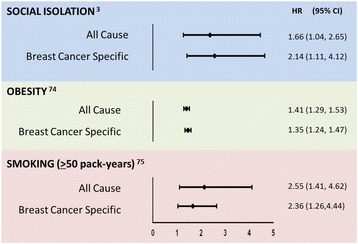
Social isolation is a risk factor for mortality on par with obesity and smoking among patients with breast cancer (BC). A plot of all-cause and BC-specific mortality from three representative studies among patients with BC who are socially isolated (relative to socially integrated), obese at time of diagnosis (relative to normal weight), or heavy smokers (relative to never having smoked). *Circles* indicate the hazard ratios (*HR*) and *horizontal lines* indicate the 95 % confidence interval provided in the representative cited paper [74, 75]

Social isolation can be defined and quantified in terms of either objective criteria, such as social network size or number/frequency of interactions with others, or subjective criteria, such as an individual’s level of perceived social isolation (colloquially, loneliness); but generally isolation refers to a complete or near-complete lack of interaction. Social environments are also assessed in terms of the level of social support provided, including both emotional support and instrumental support (help with daily tasks/transportation/treatment). The various social measures are often correlated and may share similar underlying biologic and neurologic effectors, yet concurrence on health outcomes is not 100 % among the various measures [7]; part of the difficulty in interpreting and combining conclusions from the literature arises from variation in the social measures being assessed. Importantly, social isolation is significantly associated with overall mortality in both men and women, even after correcting for demographic factors and baseline health [8], and is as strong a risk factor for morbidity and mortality as the more traditional risk factors of high blood pressure, obesity, and smoking [1]. Likewise, the risk of mortality among socially isolated patients with BC appears to be comparable to the risk conferred by obesity and smoking (Fig. 1).

The “social control theory” is often evoked to explain the effects of social isolation on human health; it posits that people with strong social networks are healthier because network members discourage detrimental health behaviors and encourage beneficial health behaviors [9]. However, the ability to recapitulate the effects of social isolation on a vast number of health outcomes in species ranging from flies to nonhuman primates suggests that an alternative explanation exists; social isolation changes an individual’s physiology in a manner that increases vulnerability to a multitude of diseases.

There are currently two leading physiological frameworks that have been developed to explain how social relationships may influence health outcomes. The “stress buffering model” posits that affiliative social interactions provide a buffer against the physiological and psychological effects of acute and chronic stressors, which in turn negatively influence health [10]; this hypothesis is supported by data indicating that the amplitude and duration of stress responses are attenuated in socially integrated animals [11]. In other words, positive social relationships have the potential to temper the deleterious effects of stress on health. Alternatively, the “main effects model” proposes that social interaction or isolation can directly influence changes in disease outcomes, with ample data demonstrating effects independent of the stress response [12]. While the frameworks are conceptually distinct, there is substantial evidence that both pathways contribute to changes in the pathophysiology of BC.

## Social influences on survival following a breast cancer diagnosis

Although relatively few BC studies have directly assessed the effects of social isolation on outcomes, converging evidence from more than a dozen generally small to medium size studies conducted over the past 20 years indicate that various forms of social engagement extend the lives of patients with BC and result in a higher quality of life (QOL) after the diagnosis [13–15]. The survival data are affirmed by three large studies that examined the relationship between social networks and emotional support on survival after BC diagnosis; specifically, women who experience pre-diagnosis social isolation have both a 66 % increase in risk of all-cause mortality and a two-fold increase in risk of BC-related mortality compared to a socially integrated cohort [3] (Fig. 1). A similar study involving younger women at a later time point after BC diagnosis reports that a larger social network size is associated with reduced all-cause, but not BC-specific, mortality [16]. However, a potential limitation of this study is that it focused on the number of social relationships, whereas the quality of close relationships is typically a better predictor of mortality among patients with BC [17]. Indeed, women with small social networks and low social support are at increased risk of BC-related mortality, whereas women with small social networks but high social support are not at increased risk [15]. In contrast, women with larger social networks but greater social burden (i.e., greater caregiving responsibilities) are at increased risk of BC-related mortality [18]. Importantly, larger social networks and greater social support also are related to better physical and mental health-related QOL and reduced BC symptoms [19]. Last, in a systematic review of the literature the evidence of an association between social support and cancer progression was strongest for breast cancer as compared to other (non-breast) cancer and mixed cancers (studies involving cancers of >1 origin) [13]. None of the BC mortality studies described included biological correlates, although several authors posited that alterations in immune and endocrine function could mediate the effects of social environment on survival following a cancer diagnosis [20, 21]. Discovering the biological explanation for social modulation of BC outcomes is a critical next step in the field.

In summary, the clinical data demonstrate a role for social environment in modifying symptomology, QOL, and mortality among BC survivors. Whether the effects of social environment are direct or indirect remains to be demonstrated conclusively; there is one early study that reported that both social involvement and stress are independently related to BC survival, although social factors did not appear to moderate the effects of stress on survival [22]. These data are intriguing, but the study requires replication because our understanding of the most critical social factors related to health has evolved substantially in the intervening sixty years since the social data were first collected. In addition, the clinical success of stress interventions that focus in part on the optimization of social support systems among patients with cancer suggests that psychologic interventions deserve further study (see review [23]). Elucidating the physiological mechanisms through which social environment influences BC may clarify why some individuals are at increased risk of disease progression or mortality, and offer the potential to exploit existing biological pathways to improve conventional treatments.

## Establishing the physiological mechanisms for social influences on breast cancer

Clinical studies can provide a wealth of evidence on correlation between biological markers and measures of social factors. However, due to the crucial role of the brain in processing social cues and coordinating complex behaviors, and the nature of the physiological changes induced by social interaction, mechanistic studies need to rely heavily on animal models for establishing causal relationships between putative biological mediators and BC outcomes. Fortunately, the effects of social isolation on wellbeing and longevity have been demonstrated in a wide range of species. In particular, rodent models of social isolation have proven highly effective at recapitulating human health outcomes that are strongly influenced by social milieu, including cardiovascular disease, cerebrovascular disease, wound healing, depression, and neuropathic pain [11]. Likewise, the detrimental effect of social isolation on mammary cancer progression has been demonstrated in rats and mice [24, 25], although no causal mechanisms have been conclusively established. Insight into the likely mechanisms underlying social influences on BC comes from converging evidence from two distinct types of scientific literature, viz., (1) social influences on physiology and (2) biological pathways known to influence BC progression. Inference is strongest for the social modulation of pathways related to stress and inflammation.

### Stress-related pathways linked to breast cancer progression and their susceptibility to social modulation

**Fig. 2:**
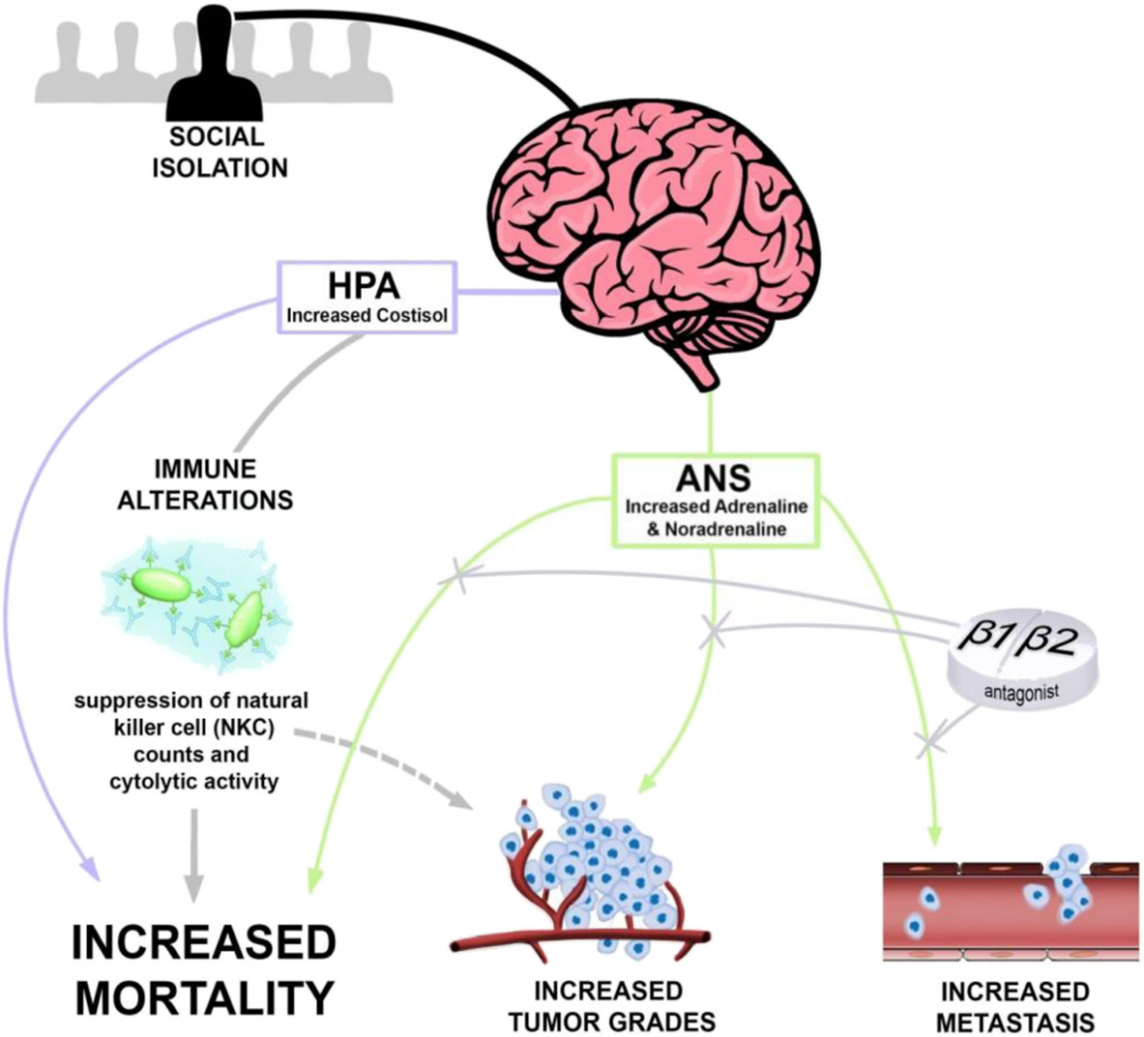
Proposed mechanisms through which social isolation influences breast cancer outcomes in women. Socially isolated individuals tend to exhibit increased activation of the hypothalamic-pituitary-adrenal (*HPA*) axis and autonomic nervous system (*ANS*). Among patients with breast cancer (BC), increased cortisol is associated with reduced natural killer cell (*NKC*) count and cytotoxicity, and increased risk of early mortality. There is also the supposition that increased concentrations of endogenous catecholamines (adrenaline and noradrenaline) promote tumor development, metastasis, and tumor progression because the use of β_1/_β_2_ receptor antagonists for other health conditions is associated with less aggressive tumors and reduced early mortality among patients with BC

All living creatures experience stress; in the short term, increased activation of the sympathetic nervous system (SNS) and hypothalamic-pituitary-adrenal (HPA) axis is typically an adaptive response to a stressor. However, as activation of these two systems becomes chronic and grows resistant to regulation, the response becomes maladaptive, increasing the individual’s susceptibility to a wide range of diseases [11]. Social isolation is a particularly potent chronic stressor for social species, and perceived isolation (loneliness) has been demonstrated in humans to correspond with increased circulating stress-related hormones [26]. Although a direct link between social environment and dysregulation of the SNS and HPA axis has not been established in patients with BC, impaired regulation of either of these endocrine systems is predictive of reduced BC-related survival [27, 28]. The primary effectors of the SNS and HPA axis are catecholamines and glucocorticoids (GCs), respectively, and these hormones are capable of modulating tumor progression. Thus, it is possible that increases in glucocorticoids and adrenergic hormones elicited via social isolation could modify breast tumor development (Fig. 2).

Converging evidence from several studies indicates that sympathetic nervous system (SNS) activation can facilitate BC progression. The SNS primarily exerts its effects through the production of adrenaline (epinephrine) from the adrenal medulla and noradrenaline (norepinephrine) from post-ganglionic neurons. In the context of cancer, most of the research to date has focused on signaling via beta-1 (β_1_) and beta-2 (β_2_) adrenergic receptors. Retrospective studies indicate that the use of β_1/_β_2_ antagonists (beta blockers) is associated with the presentation of less advanced BC, reduced metastasis, increased relapse-free survival, and reduced BC-related mortality [29–31]. A phase II clinical trial has been initiated to empirically test whether propranolol (β_1/_β_2_ antagonist) co-administration increases the efficacy of chemotherapy in patients with BC (NLM Identifier NCT01847001). There are several direct and indirect mechanisms through which β-adrenergic signaling may be influencing BC development, including tumor cell invasion [32], angiogenesis [33], tumor cell survival [29], and tumor-immune interactions [34]. Correspondingly, treatment with β-adrenergic antagonists prevents the negative effects of catecholamines on cancer growth and progression in several rodent cancer models [32, 35–37]. In addition, social isolation increases noradrenaline concentration within human ovarian tumors [38]. Together, these studies provide strong support to the hypothesis that SNS activation plays a critical role in BC progression, while offering a potential mechanism through which social isolation could be influencing BC development.

Glucocorticoids also may directly and indirectly influence tumor growth through their involvement in the fundamental biological processes of metabolism, immune function, angiogenesis, circadian rhythmicity and neuronal function. Indeed, among patients with metastatic BC, a blunted diurnal cortisol rhythm (i.e., the primary glucocorticoid in humans) is associated with reduced survival duration and the suppression of natural killer cell (NKC) counts [28]. NKCs have an innate ability to target and kill cancer cells, and higher NKC activity has been shown in patients with BC to predict disease-free survival [39] and are associated with increases in perceived levels of social support [40]. In culture, glucocorticoids promote tumor cell proliferation [41] and cancer cell survival [42]. In mice, glucocorticoids released by chronic restraint stress promote tumorigenesis through regulation of the tumor suppressor p53 [43]. Excess chronic glucocorticoids also may interfere with the efficacy of chemotherapy; both chronic stress and supplementation with exogenous glucocorticoids produce resistance to paclitaxel, in turn leading to larger tumors [44, 45]. Likewise, treatment with a glucocorticoid receptor antagonist increases the efficacy of paclitaxel-induced cytotoxicity and apoptosis in glucocorticoid-receptor-positive, triple-negative, BC cell cultures [46]. Glucocorticoids also are immunomodulators capable of altering host-tumor interaction; indeed, several immune cells that are demonstrated to play an important role in tumor development, including B cells, T cells, NKCs, and macrophages, can be modified through glucocorticoid signaling (reviewed in [47]). The effects of perceived isolation on immune function and inflammation via glucocorticoid signaling are extensive. Genome-wide expression analysis has revealed that adults with increased loneliness have upregulated expression of pro-inflammatory transcripts (associated with nuclear factor kappa B activation) and downregulated anti-inflammatory (associated with glucocorticoid receptor activation) gene expression [48]. In addition, loneliness may directly reduce the sensitivity of the glucocorticoid receptor, leading to decreased anti-inflammatory effects of glucocorticoids in isolated individuals. Loneliness is indeed associated with changes in circulating neutrophil-lymphocyte and neutrophil-monocyte ratios, both surrogate measures indicating systemic leukocyte glucocorticoid resistance in lonely individuals [49].

Interestingly, however, chronic synthetic glucocorticoid use for other health reasons was not associated with increased risk of BC or its recurrence in a large cohort of Scandinavian women [50, 51]. Furthermore, synthetic glucocorticoids (e.g., dexamethasone) are routinely used to combat the acute hypersensitivity and emetic side-effects of chemotherapy, in turn greatly improving tolerability for some patients. Whether the prophylactic use of glucocorticoids in this regard reduces the efficacy of the chemotherapy in patients, as suggested by the rodent and cell culture studies described above, is not known. To our knowledge, a systematic study of prophylactic glucocorticoid use in patients undergoing chemotherapy has not been conducted, although a clinical study examining the effects of various chemotherapy dosing regimens on disease-free and overall survival in 1572 breast cancer patients reported that dexamethasone use did not affect outcome [52]. This potential discrepancy between the effects of chronic exposure to excess endogenous versus synthetic glucocorticoids, and the direct effects of glucocorticoids on the tumor cells versus the whole body response is critical to resolve in order to optimize treatment for patients with BC. At any rate, HPA axis dysregulation of endogenous glucocorticoids provides another plausible physiological link between social isolation and BC; social isolation alters cortisol rhythms among otherwise healthy individuals [53], while disruption of diurnal cortisol rhythms is associated with a poorer prognosis among patients with BC [28].

### Social influences on mammary tumors in rodents

As described above, social influences on a wide array of health conditions, including mammary cancer can be reproduced in rodents by comparing individually housed versus pair-housed or group-housed rodents. Indeed, rodent studies have been remarkably consistent in reporting a detrimental effect of social isolation on mammary tumor progression. Specifically, social isolation in both a Sprague-Dawley rat model of spontaneous BC and a transgenic mouse model of triple-negative BC (C3(1)/SV40 Tag) results in increased tumor burden and invasiveness or malignancy [24, 25]. Interestingly, the likelihood of developing at least one tumor is comparable for both socially isolated and group-housed animals [24, 25], which mirrors the clinical data indicating that psychological factors more profoundly influence tumor growth and progression than initiation [54]. A similar, but less compelling, pattern emerges among female mice with severe immunodeficiency (SCID) injected with a human BC cell line; these mice exhibit a transient increase in tumor volume relative to group-housed mice, but only if they are isolated after the tumors become palpable [55]. One potential confounding factor in this study is that SCID mice exhibit deficits in social behavior. They do not show a preference for interacting with other mice relative to inanimate objects [56]; thus, SCID mice may not derive as great a health benefit from social interaction as more social mouse strains and other species.

**Fig. 3:**
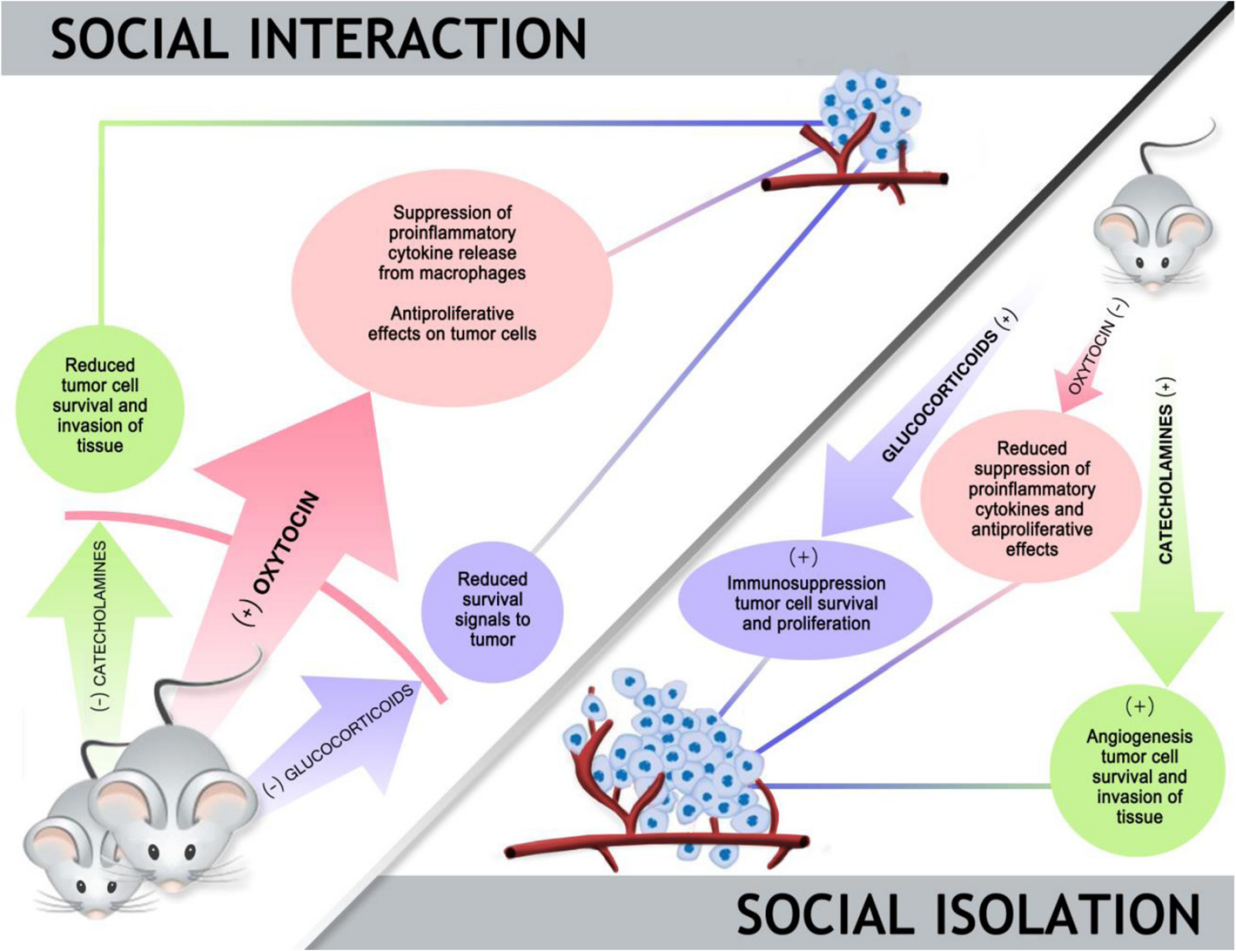
Social environment alters an animal’s hormonal milieu in a way that could either promote or suppress tumor development. Socially isolated rodents are prone to increased hypothalamic-pituitary-adrenal (HPA) axis and autonomic nervous system (ANS) activity, characterized by increased endogenous concentrations of corticosterone, adrenaline, and noradrenaline. These hormones have been shown in vivo and in vitro to promote tumor growth through a variety of well-described tumor-associated pathways. In contrast, central nervous system (CNS) oxytocin is relatively low among socially isolated animals. Among socially integrated animals there is increased release of CNS oxytocin, which in turn restrains both the HPA axis and ANS. In addition, oxytocin may indirectly influence tumor development via its modulation of macrophages

To date, none of the rodent studies demonstrating social modulation of mammary tumors have conclusively established a causal mechanism, although there are several promising leads (Fig. 3). One likely mechanism is increased corticosterone exposure among the socially isolated mice; corticosterone is the primary glucocorticoid in rodents and the magnitude and duration of corticosteroid response to a mild stressor is increased by social isolation [24, 25]. In turn, both of these aspects of the corticosteroid response are predictive of high future mammary tumor burden [24]. Accordingly, social isolation may exert some of its effects through modulation of stress pathways, as suggested by the “stress buffering” model. Furthermore, among socially isolated mice, glucocorticoid receptors in tumor cells are more likely to be localized to the nucleus than the cytoplasm, which is associated with increased resistance to chemotherapy [24]. Thus, the shift in HPA axis function that occurs in response to social isolation could potentially alter both tumor development and treatment efficacy.

Surprisingly, only one rodent study has specifically examined the role of the SNS in social modulation of mammary cancer [55]. As described above, socially isolated SCID mice injected with a human BC cell line exhibited a transient increase in tumor size that coincided with a trend toward increased norepinephrine (NE) and interleukin-6 (IL-6) in the tumor. Indeed, within days of social isolation, splenic NE and organ weight increased and there was a shift toward the augmentation of splenic macrophages that are F4/80+ and CD11b + [55]. The authors propose that the migration of these macrophage populations to the tumor would support tumor progression [55]; indeed, tumor-associated macrophages have many pro-tumoral functions, including suppression of adaptive immunity, secretion of growth factors, and stimulating angiogenesis. Whether these functions are augmented in socially isolated mice with mammary tumors remains to be determined. However, given the well-characterized effects of social isolation on SNS function, and the compelling clinical and basic science data delineating the effects of SNS activation on mammary tumors, this is an important direction for continued research.

One of the greatest advantages of using mouse models to study BC is the ability to isolate specific cellular components within the tumor microenvironment. For example, several genes involved in cancer-associated glycolysis and lipogenesis (Acetyl-CoA carboxylase alpha (Acaca), ATP citrate lyase (Acly), and Hexokinase 2 (Hk2)) are upregulated in the mammary tissue of socially isolated mice [24]. Furthermore, the social modulation of these glycolytic and lipogenic pathways is amplified in mammary adipocytes relative to non-adipocyte cell populations [57]. In addition, these metabolic changes do not exist in visceral (non-mammary) adipose cells obtained from the same isolated and group-housed cohorts [57]. Media conditioned by co-incubation with cultured adipocytes from isolated mice caused a significant increase in proliferation of a mammary cancer cell line. While there was significantly increased leptin expression and secretion from mammary adipocytes, treatment of cells with exogenous leptin did not increase proliferation, suggesting that other adipocyte-secreted factors may mediate the effects of isolation. Acaca expression has been shown to increase in adipose tissue with glucocorticoid exposure [58], offering another plausible link between social isolation and BC. Further work in identifying components of the tumor microenvironment that respond to and mediate the effects of social isolation is crucial to understanding the psychosocial effects on BC, and may yield important new therapeutic targets.

### Potential pathways identified in ovarian cancer

There are substantial biological differences among cancers originating in different tissues; however, through the analysis of shared systems and pathways in a variety of cancers, insight can be gained into possible mediators that may play a role in BC. For example, social factors have been demonstrated to influence ovarian cancer outcomes. Increased social attachment, a subtype of emotional support, has been associated with a lower likelihood of death and increased survival time in follow up of patients with diagnosed epithelial ovarian cancer [4]. However, no significant association was found between instrumental support and survival. In common with breast cancer, beta-adrenergic signaling has been identified as important in ovarian tumors, with perceived low levels of support correlated with higher norepinephrine levels within ovarian tumors, but not plasma [38]. The literature on ovarian tumors implicates a wealth of pathways related to tumor progression, including inflammatory cytokines IL-6 and IL-8 [59, 60], matrix-metalloproteinase-related tissue invasion [32], vascular endothelial growth factor (VEGF)-mediated angiogenesis [33], and NKC activity [61]. In conjunction with the BC data, these data provide converging evidence that pathways involving angiogenesis, NKC function, and inflammatory signaling may be shared mechanisms through which the social environment affects cancer progression.

### A potential role for oxytocin in social modulation of breast cancer

Oxytocin (OT) has remained understudied as a possible mediator of social influences on mammary cancer, though it is released during social and physical contact, serves important biological functions in the mammary gland, and modulates both the HPA axis and SNS. OT has a demonstrated role in the mechanisms through which social isolation affects a wide range of disease outcomes, including wound healing, pain responses, atherosclerosis, cerebral ischemia, and depressive-like behavior [11]. Additionally, OT has also been identified as a mediator of the effects of social support on buffering physiologic and behavioral stress responses [62]. However, there are few preclinical data on the effects of OT on tumor cell lines and cancer models. The data from established cancer cell lines indicate that the effects of OT are highly dependent on the origin and background of the cell line. In some studies, OT inhibits proliferation of breast cancer cells [63] while in others it increases proliferation [64].

The synthesis of OT within tumors has also been demonstrated [65], and its receptors are expressed in a variety of tumors, including those originating from the breast [66, 67]. OT receptors are present on vasculature [68], and can suppress pro-inflammatory cytokine production from macrophages [69, 70], which play a pivotal role in both tumor growth and development [71, 72]. The relative lack of data on the role of OT in mammary cancer results in part from technical challenges associated with accurately measuring OT [73]. Given that OT is modulated by social isolation and interaction, directly affects tumor cell growth, is present with its receptor in tumors, and has the ability to interact with tumor-modifying immune cells, it is a protein that warrants further exploration in BC.

## Conclusion and future challenges

In summary, social isolation is detrimental to overall health and has been identified as an exacerbating factor in many disease states. The clinical literature on BC convincingly demonstrates an association between social isolation and decreased survival following BC; several potential hormonal, angiogenic, and inflammatory markers and mediators have been identified. The critical next step will be full characterization of the physiological mechanisms underlying social influences on BC progression, including the establishment of causation for key factors. An improved understanding of the biological pathways will lead to identification of the most meaningful biomarkers and will provide context for correlations reported in clinical studies. Improved understanding of the biology also could reveal novel therapeutic targets for pharmaceutical development, identify individuals whose biology puts them at increased risk of BC recurrence or mortality, and clarify the pathways through which psychosocial interventions improve BC outcomes.

## Abbreviations

Acaca: Acetyl-CoA carboxylase alpha
Acly: ATP citrate lyase
ANS: autonomic nervous system
BC: breast cancer
CNS: central nervous system
GC: glucocorticoid
Hk2: hexokinase 2
HPA: hypothalamic-pituitary-adrenal
IL: interleukin
NE: norepinephrine
NKC: natural killer cell
OT: oxytocin
QOL: quality of life
SCID: severe combined immunodeficiency
SNS: sympathetic nervous system
